# High Seroprevalence of *Mycoplasma pneumoniae* IgM in Acute Q Fever by Enzyme-Linked Immunosorbent Assay (ELISA)

**DOI:** 10.1371/journal.pone.0077640

**Published:** 2013-10-17

**Authors:** Chung-Hsu Lai, Lin-Li Chang, Jiun-Nong Lin, Wei-Fang Chen, Li-Li Kuo, Hsi-Hsun Lin, Yen-Hsu Chen

**Affiliations:** 1 Graduate Institute of Medicine, College of Medicine, Kaohsiung Medical University, Kaohsiung City, Taiwan; 2 Division of Infectious Diseases, Department of Internal Medicine, E-Da Hospital/I-Shou University, Kaohsiung City, Taiwan; 3 Division of Infection Control Laboratory, E-Da Hospital/I-Shou University, Kaohsiung City, Taiwan; 4 Faculty of Medicine, Department of Microbiology, College of Medicine, Kaohsiung Medical University, Kaohsiung City, Taiwan; 5 Research and Diagnostic Center, Centers for Disease Control, Department of Health, Taipei, Taiwan; 6 Institute of Clinical Medicine, National Yang-Ming University, Taipei City, Taiwan; 7 Division of Infectious Diseases, Department of Internal Medicine, Kaohsiung Medical University Hospital, Kaohsiung Medical University, Kaohsiung City, Taiwan; 8 School of Medicine, College of Medicine, Kaohsiung Medical University, Kaohsiung City, Taiwan; London School of Hygiene and Tropical Medicine, United Kingdom

## Abstract

Q fever is serologically cross-reactive with other intracellular microorganisms. However, studies of the serological status of *Mycoplasma pneumoniae* and *Chlamydophila pneumoniae* during Q fever are rare. We conducted a retrospective serological study of *M. pneumoniae* and *C. pneumoniae* by enzyme-linked immunosorbent assay (ELISA), a method widely used in clinical practice, in 102 cases of acute Q fever, 39 cases of scrub typhus, and 14 cases of murine typhus. The seropositive (57.8%, 7.7%, and 0%, *p*<0.001) and seroconversion rates (50.6%, 8.8%, and 0%, *p*<0.001) of *M. pneumoniae* IgM, but not *M. pneumoniae* IgG and *C. pneumoniae* IgG/IgM, in acute Q fever were significantly higher than in scrub typhus and murine typhus. Another ELISA kit also revealed a high seropositivity (49.5%) and seroconversion rate (33.3%) of *M. pneumoniae* IgM in acute Q fever. The temporal and age distributions of patients with positive *M. pneumoniae* IgM were not typical of *M. pneumoniae* pneumonia. Comparing acute Q fever patients who were positive for *M. pneumoniae* IgM (59 cases) with those who were negative (43 cases), the demographic characteristics and underlying diseases were not different. In addition, the clinical manifestations associated with atypical pneumonia, including headache (71.2% vs. 81.4%, *p*=0.255), sore throat (8.5% vs. 16.3%, *p*=0.351), cough (35.6% vs. 23.3%, *p*=0.199), and chest x-ray suggesting pneumonia (19.3% vs. 9.5%, *p*=0.258), were unchanged between the two groups. Clinicians should be aware of the high seroprevalence of *M. pneumoniae* IgM in acute Q fever, particularly with ELISA kits, which can lead to misdiagnosis, overestimations of the prevalence of *M. pneumoniae* pneumonia, and underestimations of the true prevalence of Q fever pneumonia.

## Introduction

Q fever, a zoonosis distributed worldwide, is caused by infection with the obligate intracellular microorganism *Coxiella burnetii* [1]. The primary animal reservoirs include cattle, sheep, and goats, and the major transmission route is human inhalation of aerosols or ingestion of unpasteurized dairy products contaminated with feces, urine, or reproductive tissues of infected animals. The clinical presentation of Q fever includes acute and chronic forms [2]. Acute Q fever presents with asymptomatic infection or influenza-like symptoms with various degrees of pneumonia or hepatitis. Culture-negative infective endocarditis is the major presentation of chronic Q fever [2]. During the largest outbreak of Q fever in the Netherlands, pneumonia (61.5%) was the most common presentation, and hepatitis accounted for only 0.4% [3]. 

*Mycoplasma pneumoniae* and *Chlamydophila pneumoniae* are common atypical pathogens of community-acquired pneumonia [4]. Due to a laborious and expensive culture procedure, serological assessment of antibodies is widely used to diagnose *M. pneumoniae* [5] and *C. pneumoniae* [6], with microimmunofluorescence (MIF) as the recommended method. However, because MIF is a time-consuming procedure and requires an experienced operator, the enzyme-linked immunosorbent assay (ELISA) has become the most commonly used method in clinical practice. 

Because culture of *C. burnetii* is a laborious procedure and must be performed in a biosafety level 3 laboratory, serological assessment of antibodies against *C. burnetii* has become the gold-standard method for clinical diagnosis of Q fever. However, serological cross-reactivity with other intracellular pathogens, including *Bartonella* species [7,8], *Legionella micdadei* [9], *Rickettsia* species [10], *Chlamydophila* species [11], *Mycoplasma pneumoniae* [10], cytomegalovirus [10], and Epstein-Barr virus [10], has been reported in Q fever patients. Recently, a case of acute Q fever masquerading as *M. pneumoniae* pneumonia was reported [12]. In clinical practice, we have found several cases of acute Q fever that were serologically positive for *M. pneumoniae* or *C. pneumoniae* IgM before the final results of acute Q fever examinations were available. This finding indicates that patients can be misdiagnosed as atypical pneumonia if they are not tested for Q fever.

The aim of this study was to investigate the seroprevalence of antibodies against *M. pneumoniae* and *C. pneumoniae*, the two most common pathogens of atypical pneumonia, in patients with acute Q fever.

## Methods

### Selection of study cases

 Because acute Q fever, scrub typhus (caused by *Orientia tsutsugamushi*), and murine typhus (caused by *Rickettsia typhi*) are the most common rickettsioses in Taiwan, and because they are difficult to differentiate from each other by clinical manifestations [13], cases confirmed for the 3 diseases were included in the investigation. From April 2004 to December 2009, a total of 166 cases of acute Q fever, scrub typhus, and murine typhus were diagnosed and confirmed by the Centers for Diseases Control of Taiwan (Taiwan CDC) at E-Da hospital. Among them, the sera of 160 cases were available for study. To clarify the seroprevalence of each disease, 5 previously published cases of acute Q fever and scrub typhus co-infections were excluded [14]. Finally, the sera (acute or convalescent phase) of 155 cases (102 acute Q fever, 39 scrub typhus, and 14 murine typhus) were included in the study. Among them, 135 cases (89 acute Q fever, 34 scrub typhus, and 12 murine typhus) had paired sera (acute and convalescent phase) for investigating the rates of antibody seroconversion. 

### Ethics Statement

This study was approved by the Ethics Committee of the E-Da Hospital (EMRP-097-117). The committee waived the need for written informed consent because the demographic information and clinical data were retrospectively recorded, and all of the data were collected anonymously.

### Clinical characteristics and data collection

 The demographic information, clinical manifestations, and results of laboratory and imaging examinations of the included cases were obtained retrospectively by medical chart review and were recorded using an anonymous case record form.

### Confirmatory diagnosis of acute Q fever, scrub typhus, and murine typhus

Serological tests for the presence of specific antibodies against *C. burnetii*, *O. tsutsugamushi*, and *R. typhi* were performed using an indirect immunofluorescence antibody assay (IFA) in the contract laboratory of the Taiwan CDC, as previously described [13]. Acute Q fever was diagnosed either by an anti-phase II antigen IgG titer of ≥ 1:320 and an anti-phase II antigen IgM titer of ≥ 1:80, a four-fold or greater increase of anti-phase II antigen IgG titer in paired sera, or by blood that tested positive for *C. burnetii* DNA by polymerase chain reaction (PCR) [15]. Scrub typhus and murine typhus were diagnosed by a specific antibody titer of IgM ≥ 1:80, a four-fold or greater rise of IgG titer in paired sera, or by blood that tested positive for *O. tsutsugamushi* and *R. typhi* DNA by PCR, respectively. 

### Detection of serum antibodies against *Mycoplasma pneumoniae* and *Chlamydophila pneumoniae*

 Serum was the residual specimen obtained for the diagnosis-related purposes of rickettsioses for the Taiwan CDC, and it was stored at -80°C until analysis. The serum IgG and IgM antibodies against *M. pneumoniae* and *C. pneumoniae* were detected using commercially available ELISA kits of *M. pneumoniae* IgG (MYCG0350)/IgM (MYCM0350) and *C. pneumoniae* IgG (CHLG0510)/IgM (CHLM0510) (ELISA kits, NovaLisa™, NovaTec Immundiagnostica GmbH, Germany), respectively. These kits were used for the detection of serum *M. pneumoniae* and *C. pneumoniae* IgG or IgM in clinical practice at E-Da Hospital. To validate the results, a second commercial ELISA kit for the detection of *M. pneumoniae* IgG (SeroMP^TM^ IgG)/IgM (SeroMP^TM^ IgM) and *C. pneumoniae* IgG (SeroCP^TM^ IgG)/IgM (SeroCP^TM^ IgM) (ELISA kits, Savyon Diagnostics, Ashdod, Israel) was used. All of the procedures and the interpretation of antibody determinations were performed according to the manufacturer's instructions, and each examination was performed in duplicate.

### Statistical analysis

Categorical variables were analyzed using the Chi-square or Fisher’s exact test where appropriate. Continuous variables were analyzed using Student’s t-test. All *p* values were two-tailed, and a *p* value <0.05 was considered to be statistically significant. The data were analyzed with SPSS software for Windows (Release 15.0; SPSS, Chicago, IL).

## Results

### Overall status of *M. pneumoniae* and *C. pneumoniae* IgG/IgM in acute Q fever, scrub typhus, and murine typhus

**Figure 1:**
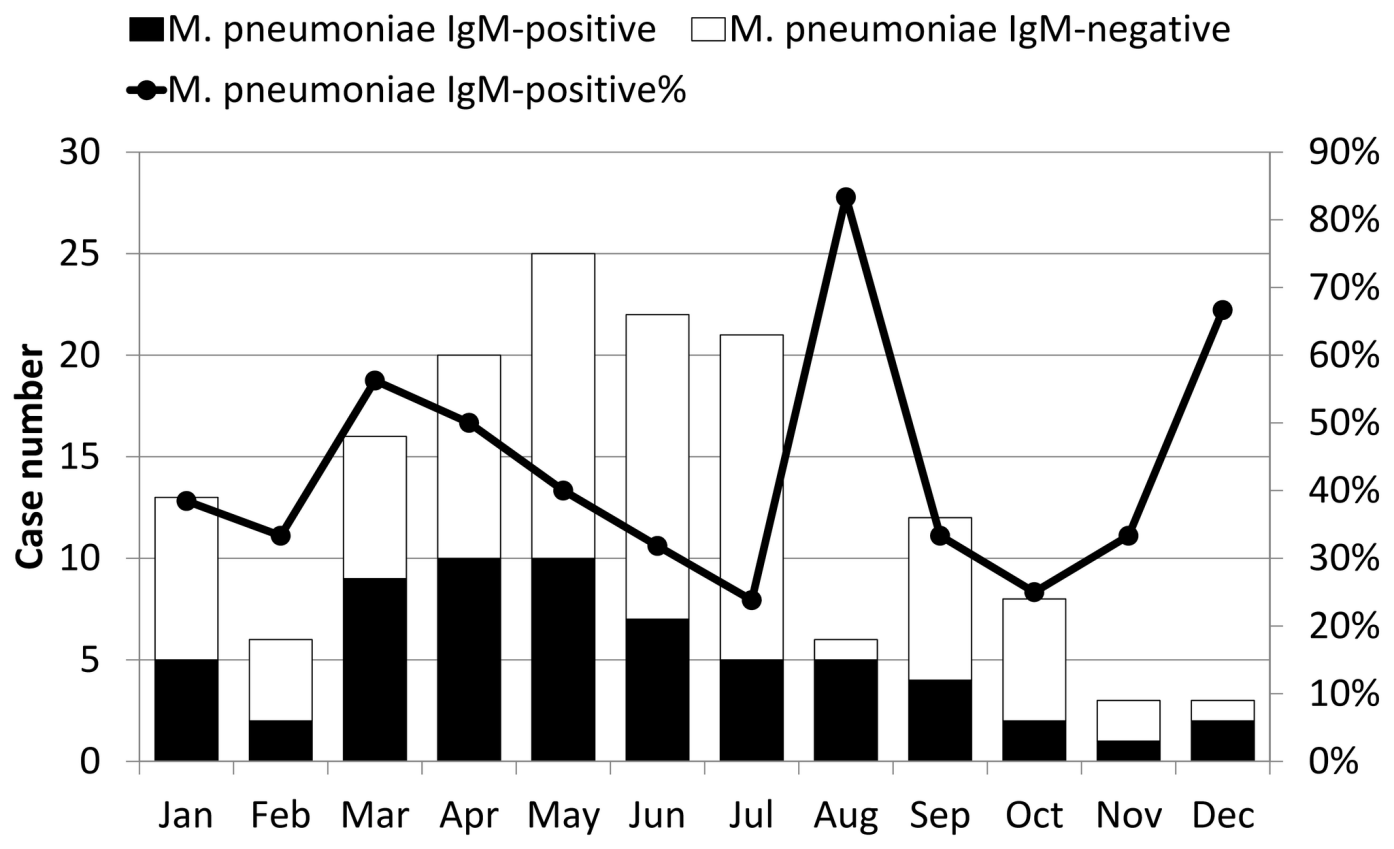
Monthly distribution of 62 cases with either acute or convalescent phase *Mycoplasma pneumoniae* IgM sera, 155 cases of acute Q fever (59 of 102 cases, 57.8%), scrub typhus (3 of 39 cases, 7.7%), and murine typhus (0 of 14 cases, 0%).

**Figure 2:**
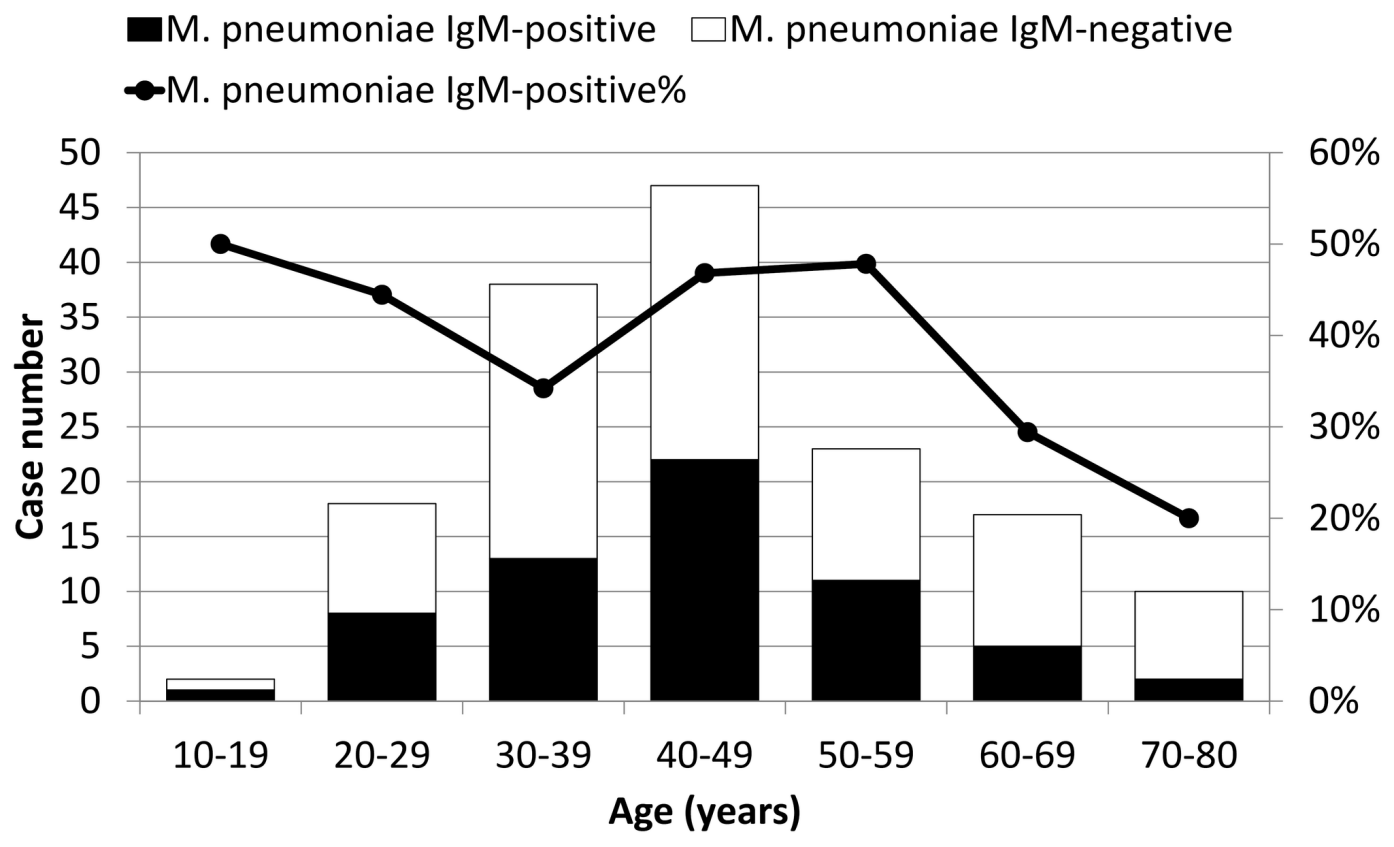
Age distribution of 62 cases with either acute or convalescent phase *Mycoplasma pneumoniae* IgM sera, 155 cases of acute Q fever (59 of 102 cases, 57.8%), scrub typhus (3 of 39 cases, 7.7%), and murine typhus (0 of 14 cases, 0%).

The serum IgG or IgM results of *M. pneumoniae* and *C. pneumoniae* in the 155 cases using the first ELISA kits are shown in Table 1. In acute or convalescent phase sera, 62 (40.0%) and 43 cases (27.7%) had *M. pneumoniae* IgM and *C. pneumoniae* IgM, respectively. The monthly distribution of serum positive cases of *M. pneumoniae* IgM is shown in Figure 1, and they reached peaks in March, August, and December. Figure 2 illustrates the age distribution of the serum positive cases of *M. pneumoniae* IgM, and it was highest for those aged between 40 and 59 years old. The positivity rates of *M. pneumoniae* IgM in the acute (13.9%, 0%, and 0%, *p*=0.012), convalescent (58.9%, 8.8%, and 0%, *p*<0.001), and acute or convalescent phases (57.8%, 7.7%, and 0%, *p*<0.001) of acute Q fever were significantly higher than scrub typhus or murine typhus (Table 1). In contrast, the positivity rates of *M. pneumoniae* IgG and *C. pneumoniae* IgG/IgM were not different between the 3 diseases. To further investigate the seroconversion of *M. pneumoniae* and *C. pneumoniae* antibodies at the acute and convalescent phases, the 135 patients with available paired sera were analyzed, and the results are shown in Table 2. The seroconversion rate of *M. pneumoniae* IgM in acute Q fever was significantly higher than scrub typhus or murine typhus (50.6%, 8.8%, and 0%, *p*<0.001). However, the seroconversion rates of *M. pneumoniae* IgG and *C. pneumoniae* IgG/IgM were not different. Among the 56 acute Q fever patients with serum *M. pneumoniae* IgM, only 2 (3.6%) and 16 (28.6%) had 4-fold increases of IgG and IgG seroconversion, respectively (Table 3). Taken together, only the positivity and seroconversion rates of *M. pneumoniae* IgM in acute Q fever were significantly higher than scrub typhus or murine typhus.

**Table 1:** The results of all available sera tested for *Mycoplasma pneumoniae* and *Chlamydophila pneumoniae* IgM and IgG antibodies^**a**^ in patients with acute Q fever, scrub typhus, and murine typhus.

Positive antibodies in the acute or convalescent phase	Acute Q fever (N=102)	Scrub typhus (N=39)	Murine typhus (N=14)	Total (n=155)	*p* ^f^
*Mycoplasma pneumoniae* IgM^b^					
Acute or convalescent phase	59/102 (57.8)	3/39 (7.7)	0/14 (0)	62/155 (40.0)	<0.001
Acute phase	14/101 (13.9)	0/39 (0)	0/13 (0)	14/153 (9.2)	0.012
Convalescent phase	53/90 (58.9)	3/34 (8.8)	0/13 (0)	56/137 (40.9)	<0.001
*Mycoplasma pneumoniae* IgG^c^					
Acute or convalescent phase	72/102 (70.6)	22/39 (56.4)	9/14 (64.3)	103/155 (66.5)	0.274
Acute phase	51/101 (50.5)	14/39 (35.9)	6/13 (46.2)	71/153 (46.4)	0.308
Convalescent phase	63/90 (70.0)	17/34 (50.0)	7/13 (53.8)	87/137 (63.5)	0.092
*Chlamydophila pneumoniae* IgM^d^					
Acute or convalescent phase	26/102 (25.5)	11/39 (28.2)	6/14 (42.9)	43/155 (27.7)	0.357
Acute phase	11/101(10.9)	6/39 (15.4)	2/13 (15.4)	19/153 (12.4)	0.671
Convalescent phase	22/90 (24.4)	10 /34(29.4)	6/13 (46.2)	38/137 (27.7)	0.279
*Chlamydophila pneumoniae* IgG^e^					
Acute or convalescent phase	67/102 (65.7)	31/39 (79.5)	11/14 (78.6)	109/155 (70.3)	0.257
Acute phase	47/101 (46.5)	23/39 (59.0)	8/13 (61.5)	78/153 (51.0)	0.326
Convalescent phase	55/90 (61.1)	26/34 (76.5)	10/13 (76.9)	91/137 (66.4)	0.197

a Tested by ELISA (NovaTec Immundiagnostica GmbH, Germany).

b Positive threshold for *M. pneumoniae* IgM is > 11 NTU. The positive values ranged from 11.3 to 46.9 (median=15.6) NTU.

c Positive threshold for *M. pneumoniae* IgG is > 11 NTU. The positive values ranged from 11.1 to 47.9 (median=17.0) NTU.

d Positive threshold for *C. pneumoniae* IgM is > 11 NTU (NovaTec-Units). The positive values ranged from 11.1 to 36.2 (median=15.7) NTU.

e Positive threshold for *C. pneumoniae* IgG is > 11 NTU. The positive values ranged from 11.1 to 63.1 (median=20.3) NTU.

f Chi-square or Fisher’s exact test between acute Q fever, scrub typhus, and murine typhus.

**Table 2:** The results of paired sera tested for *Mycoplasma pneumoniae* and *Chlamydophila pneumoniae* IgM and IgG antibodies^**a**^ in patients with acute Q fever, scrub typhus, and murine typhus.

Antibodies in the acute and convalescent phase	Acute Q fever (N=89)	Scrub typhus (N=34)	Murine typhus (N=12)	Total (N=135)	*p* ^f^
*Mycoplasma pneumoniae* IgM^b^					
Seroconversion	45 (50.6)	3 (8.8)	0 (0)	48 (35.6)	<0.001
Seroreversion	3 (3.4)	0 (0)	0 (0)	3 (2.2)	0.668
Both positive	8 (9.0)	0 (0)	0 (0)	8 (5.9)	0.151
Both negative	33 (37.1)	31 (91.2)	12 (100)	76 (56.3)	<0.001
*Mycoplasma pneumoniae* IgG^c^					
Seroconversion	21 (23.6)	8 (23.5)	2 (16.7)	31 (23.0)	0.950
Seroreversion	1 (1.1)	2 (5.9)	2 (16.7)	5 (3.7)	0.018
Both positive	42 (47.2)	9 (26.5)	4 (33.3)	55 (40.7)	0.093
Both negative	25 (28.1)	15 (44.1)	4 (33.3)	44 (32.6)	0.245
*Chlamydophila pneumoniae* IgM^d^					
Seroconversion	15 (16.9)	5 (14.7)	4 (33.3)	24 (17.8)	0.372
Seroreversion	3 (3.4)	1 (2.9)	0 (0)	4 (3.0)	0.999
Both positive	7 (7.9)	5 (14.7)	2 (16.7)	14 (10.4)	0.313
Both negative	64 (71.9)	23 (67.6)	6 (50.0)	93 (68.9)	0.287
*Chlamydophila pneumoniae* IgG^e^					
Seroconversion	19 (21.3)	8 (23.5)	2 (16.7)	29 (21.5)	0.947
Seroreversion	7 (7.9)	3 (8.8)	0 (0)	10 (7.4)	0.776
Both positive	35 (39.3)	18 (52.9)	7 (58.3)	60 (44.4)	0.240
Both negative	28 (31.5)	5 (14.7)	3 (25.0)	36 (26.7)	0.160

a Tested by ELISA (NovaTec Immundiagnostica GmbH, Germany).

b Positive threshold for *M. pneumoniae* IgM is > 11 NTU (NovaTec-Units). The positive values ranged from 11.3 to 46.9 (median=15.8) NTU.

c Positive threshold for *M. pneumoniae* IgG is > 11 NTU. The positive values ranged from 11.1 to 47.9 (median=17.3) NTU.

d Positive threshold for *C. pneumoniae* IgM is > 11 NTU. The positive values ranged from 11.1 to 36.2 (median=15.8) NTU.

e Positive threshold for *C. pneumoniae* IgG is > 11 NTU. The positive values ranged from 11.1 to 63.1 (median=21.3) NTU.

f Chi-square or Fisher’s exact test between acute Q fever, scrub typhus, and murine typhus.

**Table 3:** Four-fold increases and seroconversion of *M. pneumoniae* IgG in acute Q fever with serum *M. pneumoniae* IgM by 2 ELISA kits.

ELISA kit	4-fold increase of *M. pneumoniae* IgG	Seroconversion of *M. pneumoniae* IgG
NovaTec Immundiagnostica GmbH, Germany	3.6% (2/56)^a^	28.6% (16/56)^b^
Savyon Diagnostics, Ashdod, Israel	11.1% (5/45)^c^	11.1% (5/45)^c^

a One of the two patients had seroconversion of *M. pneumoniae* IgG

b None of the 16 patients had 4-fold increases of *M. pneumoniae* IgG

c All of the 5 patients had 4-fold increases and seroconversion of *M. pneumoniae* IgG

### Results of *M. pneumoniae* and *C. pneumoniae* IgG/IgM in acute Q fever with the second ELISA kit

 The second ELISA kits (Savyon Diagnostics, Ashdod, Israel) were used to validate the high seropositivity rate of *M. pneumoniae* IgM found in acute Q fever cases. The results and concordant rates compared to the first ELISA kits are listed in Table 4. These revealed positivity rates of 21% (21/100), 45.3% (39/86), and 49.5% (51/103) during the acute, convalescent, and acute or convalescent phases, respectively. Among the cases with available paired sera, the seroconversion rate of *M. pneumoniae* IgM was 33.3% (28/84). Various degrees of concordant rates were found when compared to the first ELISA kits. The 4-fold increase of IgG and IgG seroconversion in the 45 acute Q fever patients with serum *M. pneumoniae* IgM were both 11.1% (Table 3). 

**Table 4:** The results and concordant rates of *Mycoplasma pneumoniae* and *Chlamydophila pneumoniae* IgM and IgG antibodies in patients with acute Q fever tested by ELISA (Savyon Diagnostics, Ashdod, Israel).

Antibodies in the acute and/or convalescent phase	Positive number/ tested number (%)	Concordant rates^e^ compared with ELISA kits (NovaTec Immundiagnostica GmbH, Germany)
*Mycoplasma pneumoniae* IgM^a^		
Acute phase	21/100 (21)	52.4%
Convalescent phase	39/86 (45.3)	79.5%
Acute or convalescent phase	51/103 (49.5)	72.5%
Seroconversion	28/84 (33.3)	67.9%
*Mycoplasma pneumoniae* IgG^b^		
Acute phase	18/99 (18.2)	94.4%
Convalescent phase	47/89 (52.8)	87.2%
Acute or convalescent phase	50/102 (49.0)	88.0%
Seroconversion	30/86 (34.9)	30.0%
*Chlamydophila pneumoniae* IgM^c^		
Acute phase	9/97 (9.3)	55.6%
Convalescent phase	29/86 (33.7)	27.6%
Acute or convalescent phase	34/101 (33.7)	38.2%
Seroconversion	23/82 (28.0)	21.7%
*Chlamydophila pneumoniae* IgG^d^		
Acute phase	57/98 (58.2)	73.7%
Convalescent phase	52/82 (63.4)	78.8%
Acute or convalescent phase	73/100 (73.0)	78.1%
Seroconversion	14/80 (17.5)	35.7%

a Positive threshold for *M. pneumoniae* IgM is > 20 BU/ml). The positive values ranged from 20.6 to 146.3 (median=40.7) BU/ml.

b Positive threshold for *M. pneumoniae* IgG is > 20 BU/ml. The positive values ranged from 20.1 to 163.8 (median=38.7) BU/ml.

c Positive threshold for *C. pneumoniae* IgM is > 1.5 COI. The positive values ranged from 1.51 to 5.25 (median=2.0) COI.

d Positive threshold for *C. pneumoniae* IgG is > 1.1 COI. The positive values ranged from 11.1 to 6.31 (median=2.06) COI.

e Concordant rate = number of positive samples by both kits/number of positive samples by each ELISA kit (Savyon Diagnostics, Ashdod, Israel) x 100

### Status of *M. pneumoniae* IgM and clinical manifestations of acute Q fever

 To investigate the possible clinical differences between acute Q fever with and without serum *M. pneumoniae* IgM, the 102 cases of acute Q fever listed in Table 1 were divided into 2 groups according to the results of the *M. pneumoniae* IgM in acute or convalescent phase serum for comparison (59 positive cases [57.8%] and 43 negative cases [42.2%]). There were no significant differences among demographic data, underlying diseases, or clinical symptoms and signs between the 2 groups of patients (Table 5). It is noteworthy that symptoms possibly associated with atypical pneumonia, such as headache (71.2% vs. 81.4%, *p*=0.255), sore throat (8.5% vs. 16.3%, *p*=0.351), and cough (35.6% vs. 23.3%, *p*=0.199), were not different. In imaging findings, laboratory examinations, and responses to treatment, there were no differences between the patients with and without serum *M. pneumoniae* IgM (Table 6). The rate of chest x-ray abnormalities, possibly suggesting pneumonia, was likewise unchanged (19.3% vs. 9.5%, *p*=0.258). Similarly, no significant differences were found by comparing the same variables listed in Tables 5 and 6 between patients with and without seroconversion of *M. pneumoniae* IgM (data not shown).

**Table 5:** Differences among demographic data, underlying diseases, and clinical symptoms and signs between acute Q fever patients whose sera were positive and negative for *Mycoplasma pneumoniae* IgM^a^.

Clinical characteristics	Negative for *M. pneumoniae* IgM (N=43)	Positive for *M. pneumoniae* IgM (N=59)	Total (N=102)	*p* ^b^
Demographic data and underlying diseases				
Male gender	40 (93.0)	55(93.2)	95 (93.1)	0.999
Age (years)^c^	43.6 ± 11.8	44.8 ± 12.0	44.3 ± 11.9	0.602
HBV or HCV infection^d^	11 (25.6)	21 (35.6)	32 (31.4)	0.388
Liver cirrhosis	1 (2.3)	0 (0)	1 (1.0)	0.422
Hypertension	4 (9.3)	7 (11.9)	11 (10.8)	0.757
Diabetes mellitus	4 (9.3)	3 (5.1)	7 (6.9)	0.451
Congestive heart failure	0 (0)	1 (1.7)	1 (1.0)	0.999
COPD	0 (0)	1 (1.7)	1 (1.0)	0.999
Malignancy	0 (0)	2 (3.4)	2 (2.0)	0.507
HIV infection	1 (2.3)	0 (0)	1 (1.0)	0.422
Clinical symptoms and signs				
Fever	42 (97.7)	59 (100)	101 (99.0)	0.422
Chills	34 (79.1)	50 (84.7)	84 (82.4)	0.600
Headache	35 (81.4)	42 (71.2)	77 (75.5)	0.255
Sore throat	7 (16.3)	5 (8.5)	12 (11.8)	0.351
Jaundice	2 (4.7)	3 (5.1)	5 (4.9)	0.999
Cough	10 (23.3)	21 (35.6)	31 (30.4)	0.199
Nausea or vomiting	2 (4.7)	4 (6.8)	6 (5.9)	0.999
Abdominal pain or discomfort	7 (16.3)	6 (10.2)	13 (12.7)	0.384
Diarrhea	4 (9.3)	5 (8.5)	9 (8.8)	0.999
General weakness	8 (18.6)	5 (8.5)	13 (12.7)	0.145
Arthralgia	1 (2.3)	2 (3.4)	3 (2.9)	0.999
Myalgia	19 (44.2)	18 (30.5)	37 (36.3)	0.211
Relative bradycardia^e^	16 (37.2)	32 (54.2)	48 (47.1)	0.110

a Tested by ELISA (NovaTec Immundiagnostica GmbH, Germany).

b Categorical variables were analyzed using the Chi-square or Fisher’s exact test as appropriate. Continuous variables were analyzed using Student’s t-test.

c Presented as the mean value ± standard deviation.

d Confirmed by examinations of HBsAg and anti-HCV.

e Body temperature ≥ 38.9^o^C and heart rate < 110/min without medication with calcium blockers, beta-blockers, or anti-arrhythmic agents.

**Table 6:** Differences among imaging findings, laboratory examinations, and responses to treatment between acute Q fever patients whose sera were positive and negative for *Mycoplasma pneumoniae* IgM^a^.

Clinical characteristics	Negative for *M. pneumoniae* IgM (N=43)	Positive for *M. pneumoniae* IgM (N=59)	Total (N=102)	*p* ^b^
Abdominal sonography or computed tomography findings	39 (90.7)	56 (94.9)		0.451
Days from disease onset^c^	6.8 ± 4.0	7.0 ± 3.3	6.9 ± 3.6	0.778
Abnormal	18/39 (46.2)	25/56 (44.6)	43/95 (45.3)	0.999
Hepatomegaly or splenomegaly	15 (38.5)	18 (32.1)	33/95 (34.7)	0.662
Hepatomegaly	9/39 (23.1)	13/56 (23.2)	22/95 (23.2)	0.999
Splenomegaly	12/39 (30.8)	10/56 (17.9)	22/95 (23.2)	0.216
Cholecystitic change	6/39 (15.4)	17/56 (30.4)	23/95 (24.2)	0.143
Fatty liver	22/39 (56.4)	28/56 (50.0)	50/95 (52.6)	0.676
Chest x-ray				
Days from disease onset^c^	5.3 ± 3.1	5.7 ± 2.7	5.5 ± 2.9	0.519
Abnormal CXR finding	4/42 (9.5)	11/57 (19.3)	16/99 (15.2)	0.258
Unilateral infiltration	2/42 (4.8)	5/57 (8.8)	7/99 (7.1)	0.695
Bilateral infiltration	2/42 (4.8)	6/57 (10.5)	8/99 (8.1)	0.461
Pneumonia patch	0/42 (0)	0/57 (0)	0/99 (0)	NC
Complete blood cell examination				
Days from disease onset^c^	5.1 ± 3.1	5.3 ± 2.5	5.2 ± 2.7	0.813
Leukocytosis	2 (4.7)	2 (3.4)	4/102 (3.9)	0.999
Leukopenia	9 (20.9)	7 (11.9)	16/102 (15.7)	0.273
Anemia	0 (0)	0 (0)	0/102 (0)	NC
Thrombocytopenia	30 (69.8)	43 (72.9)	73/102 (71.6)	0.825
Liver transaminase				
Days from disease onset^c^	5.6 ± 3.9	5.7 ± 2.6	5.6 ± 3.2	0.849
GPT > 88	23/42 (54.8)	42/59 (71.2)	65/101 (64.4)	0.097
GOT > 76	27/42 (64.3)	43/58 (74.1)	70/100 (70.0)	0.377
GPT > 44	40/42 (95.2)	58/59 (98.3)	98/101 (97.0)	0.569
GOT > 38	41/42 (97.6)	57/58 (98.3)	98/100 (98.0)	0.999
Doxycycline treatment to defervescence > 3 days	5/35 (14.3)	10/44 (22.7)	15/79 (19.0)	0.398

a Tested by ELISA (NovaTec Immundiagnostica GmbH, Germany).

b Categorical variables were analyzed using the Chi-square or Fisher’s exact test as appropriate. Continuous variables were analyzed using Student’s t-test.

c Presented as the mean value ± standard deviation.

## Discussion

Various degrees of sensitivity, specificity, and cross-reactivity were reported in several commercially available kits of *M. pneumoniae* [16-19] and *C. pneumoniae* [20,21], but the sera from rickettsioses were rarely used as controls for the evaluation of specificity [19]. In the present study, 57.8% and 25.5% of acute Q fever, 7.7% and 28.2% of scrub typhus, and 0% and 42.9% of murine typhus cases were serum positive for *M. pneumoniae* IgM and *C. pneumoniae* IgM, respectively (Table 1). The seropositive rates of *M. pneumoniae* IgM in acute Q fever were much higher than the acute infection rates of *M. pneumoniae* as determined by serological studies in healthy adolescents (6.0%) [22], adults with respiratory symptoms (3.3%) [23], and community-acquired pneumonia (CAP) (14.3%-20.0%) [24,25] in Taiwan. The seropositivity rates of *C. pneumoniae* IgM were also higher than the percentage of *C. pneumoniae* pneumonia in CAP in Taiwan (7.1%-13.0%) [24,25]. Accordingly, the high seropositivity rates of *M. pneumoniae* and *C. pneumoniae* IgM in the present study did not result from background seroprevalence. These results had two important impacts. First, in clinical practice, rickettsioses may be misdiagnosed as atypical pneumonia if only *M. pneumoniae* and *C. pneumoniae* antibodies are tested. Second, in the investigation of *M. pneumoniae* and *C. pneumoniae* ELISA kits, the sera from rickettsioses should be included for evaluating specificity and possible false-positivity of the ELISA kits.

Both Q fever pneumonia and *M. pneumoniae* pneumonia belong to atypical pneumonia, but serological studies for one in the other are rare [10,12,19]. A case of Q fever presented with acalculous cholecystitis was reported to have serum *M. pneumoniae* IgM (SeroMP^TM^) [12]. Vardi et al. reported that 4 of 33 (12.1%) acute Q fever cases had serum *M. pneumoniae* IgM [10]. In a study by Beersma et al., 3 of the 12 serological kits for *M. pneumoniae* had false positivity for *M. pneumoniae* IgM in 4 Q fever controls (ImmunoCard^TM^ [75%, 3/4], Novum^TM^ [100%, 4/4], SeroMP^TM^ [50%, 2/4]) [19]. In the clinical setting, possible dual infections of *C. burnetii* and *M. pneumoniae* or *C. pneumoniae* have rarely been reported in studies of CAP [26,27]. In an 11-year study conducted in the Canary Islands, 10% of CAP cases were mixed infections. *C. burnetii* was the most frequently isolated co-pathogen, but the pathogens were not clearly described [26]. In a study in northern Israel, Shibli et al. identified Q fever as the microbiological etiology of 8 CAP cases in which 3 (37.5%) and 2 (25.0%) were combined infections with *C. pneumoniae* and *M. pneumoniae*, respectively [27]. In clinical interpretations, the seropositivity and seroconversion of *M. pneumoniae* IgM suggests a recent infection, which indicates that as many as 50-60% acute Q fever cases could be diagnosed as *M. pneumoniae* infection without testing for Q fever. From the epidemiological perspective, this would underestimate and overestimate the true prevalence of Q fever and *M. pneumoniae* infections, respectively.

Acute Q fever, followed by scrub typhus and murine typhus, is the most common rickettsiosis, and it is an emerging and endemic disease in southern Taiwan [13]. In Taiwan, hepatitis rather than pneumonia is the predominant presentation of acute Q fever [28,29]. In our previous study, although all of the cases of acute Q fever had hepatitis, 19% of them had abnormal chest x-ray findings, and 9.5% had unilateral and 9.5% bilateral infiltration [29]. This result indicated that nearly 20% of acute Q fever cases might be diagnosed and treated as pneumonia based on chest x-ray findings without testing for Q fever. In the present study, 57.8% and 25.5% of acute Q fever cases had serum *M. pneumoniae* IgM and *C. pneumoniae*, respectively (Table 1), and 19.3% of cases with *M. pneumoniae* IgM had abnormalities suggestive of pneumonia by chest x-ray (Table 6). These findings highlight that, before the confirmatory results of Q fever are available or without testing for Q fever, approximately 60% and 25% of cases may be misdiagnosed as *M. pneumoniae* and *C. pneumoniae* infection, respectively. Conversely, acute Q fever may account for a portion of serologically diagnosed *M. pneumoniae* and *C. pneumoniae* pneumonia, and the true prevalence of acute Q fever presenting with pneumonia in southern Taiwan may be underestimated.

Several points suggest that the high seropositivity rate of *M. pneumoniae* IgM in acute Q fever may be due to serological cross-reactivity rather than true *M. pneumoniae* infection. First, in addition to developing auto-antibodies [10,30,31], serological cross-reactivity with other pathogens is common in Q fever, including *M. pneumoniae* [7-11]. Second, the risk of dual infections of non-zootic (*M. pneumoniae*) and zoonotic (*C. burnetii*) atypical pneumonia is low [32]. Third, the monthly and age distributions (Figures 1 and 2) were not typical for *M. pneumoniae* pneumonia, which generally predominates in autumn among children and young adults [33]. Fourth, the clinical symptoms and chest-x ray findings possibly associated with *M. pneumoniae* pneumonia were not different between acute Q fever patients with and without serum *M. pneumoniae* IgM (Tables 5 and 6). Fifth, it is unreasonable that patients with acute Q fever rather than scrub typhus or murine typhus tend to contract dual infections with *M. pneumoniae*. From the viewpoint of the immune response to *M. pneumoniae*, despite high seropositivity (57.8%) (Table 1) and seroconversion rates of *M. pneumoniae* IgM (50.6%) (Table 2) in acute Q fever, a high IgG seropositivity rate during the acute phase (50.5%), a low IgG seroconversion rate (23.6%), and various concordant rates between the 2 ELISA kits (Table 4) are not typical serological patterns of acute *M. pneumoniae* infection. Further investigation of acute Q fever patients with *M. pneumoniae* IgM revealed that few had 4-fold increases (3.6% and 11.1%) and IgG seroconversion (28.6% and 11.1%) as measured by the two ELISA kits (Table 3), indicating that the presence of IgM did not result from true infection. However, the hypothesis of cross-reactivity between acute Q fever serum and *M. pneumoniae* IgM ELISA kits requires determination by cross-adsorption studies and Western blot analysis, which require culture and large quantities of *C. burnetii* and *M. pneumoniae* antigens in a professional research laboratory [7,9,34].

This retrospective clinical and serological study has several limitations. We only tested 2 ELISA kits commonly used in Taiwan for the serological diagnosis of *M. pneumoniae* and *C. pneumoniae* infections. Whether a similar phenomenon exists with other kits needs further investigation. The studied cases were mainly distributed across southern Taiwan, and the results require a large-scale study involving other countries to be confirmed. The hypothesis of serological cross-reactivity derived from clinical observations needs further investigation by basic medical research in a professional laboratory.

In conclusion, nearly 60% of acute Q fever cases were serum positive for *M. pneumoniae* IgM by ELISA. However, their clinical manifestations were not different from those that were serum negative for *M. pneumoniae* IgM. In addition, approximately 25% of acute Q fever cases were serum positive for *C. pneumoniae* IgM. Clinicians should be aware of the high seroprevalence of *M. pneumoniae* IgM, particularly using ELISA kits, in acute Q fever. This observation could lead to misdiagnosis, overestimation of the prevalence of *M. pneumoniae* infection, and underestimations of the true prevalence of Q fever pneumonia.
